# Negative Pressure Wound Therapy: Significant Improvement in Definitive Fascial Closure With Reduced Theatre Episodes and Complications in Patients With Encapsulating Peritoneal Sclerosis

**DOI:** 10.7759/cureus.91519

**Published:** 2025-09-03

**Authors:** Aidan Bannon, Rebecca Varley, Robert Leatherby, Titus Augustine, Zia Moinuddin, David van Dellen

**Affiliations:** 1 Department of Renal Transplantation, Manchester University NHS Foundation Trust, Manchester, GBR

**Keywords:** abdominal wall, abthera, hernia, npwt, open abdomen

## Abstract

Encapsulating peritoneal sclerosis (EPS) is a rare condition characterised by recurrent episodes of intestinal obstruction and perforation, usually due to prolonged peritoneal dialysis exposure, resulting in abdominal catastrophes often requiring open abdomen management (OAM). Dynamic negative pressure wound therapies (NPWTs) can facilitate definitive closure in the open abdomen, but consensus recommendations lack high-quality, cohort-specific data. We performed a retrospective analysis on the use of NPWTin this patient cohort to assess its effectiveness when compared to management with static management of the open abdomen.

Primary endpoints assessed were: (i) definitive closure; (ii) time to definitive closure; and (iii) method of closure. Secondary endpoints included assessment of complications. Multiple linear and logistic regression analysis assessed variables predictive of primary endpoints.

99 patients were included. 43 (43%) patients were managed with NPWT and 56 (57%) patients with static mechanisms (betadine-soaked gauze.) Patients who were managed with NPWTwere more likely to achieve fascial closure (n=27 vs n=7, p<0.0001), required less total theatre episodes (n=2.27 vs 4.78, p<0.0001), and reported less failure to close episodes (p=0.002). The use of NPWTwas associated with fewer returns to theatre following closure within 30 days (n=4 vs n=19, p=0.004). Failure to close was associated with all-cause mortality (p<0.026).

This study demonstrates that NPWT is associated with increased likelihood of fascial closure with a reduced complication profile in patients with EPS, representing a chronically malnourished, high-risk surgical cohort. NPWTshould be within the armamentarium of the general surgeon faced with a complex open abdomen and can be safely used in high-risk surgical patients.

## Introduction

Encapsulating peritoneal sclerosis (EPS) is rare and life-threatening clinical condition characterised by the encasement or cocooning of the bowel by a thickened fibrocollagenous peritoneal membrane resulting in recurrent episodes of obstruction and perforation [1-3]. This chronic condition has an insidious onset, most commonly associated with peritoneal dialysis with a prevalence of 0.4-8.9%, increasing to 20% after eight years of peritoneal dialysis [3]. EPS mortality is highest in the first year after diagnosis, ranging from 10-55% with a proportional rise in mortality relative to duration of peritoneal dialysis therapy [1,4].

Treatment for EPS has evolved over the last decade. Surgery is considered definitive management for symptomatic patients and is defined by laparotomy, enterolysis, and peritonectomy. Despite this, recurrence rates are as high as 25% [5]. Mortality associated with surgery is more common in late-stage EPS and ranges from 19-35%, but improved outcomes have been observed with improvements in surgical techniques and earlier diagnosis [3,5,6]. Surgery in this malnourished patient cohort with concomitant chronic disease increases the risk of complications, including gross postoperative ileus, tissue and small bowel oedema, haemorrhage, enterotomy, and enterocutaneous or entero-enteric fistulation. Many patients are, therefore, elected to be managed with an open abdomen, and relook is planned after 24-48 hours to provide an opportunity for a definitive decision with respect to the option of diverting stoma, further bowel resection, or ongoing laparostomy management.

Open abdomen management (OAM) is an accepted standard of treatment in damage control laparotomies for conditions including severe trauma, haemorrhage and peritonitis with organ dysfunction [7]. Consensus recommendations regarding OAM and temporary abdominal closure with dynamic negative pressure wound therapy (NPWT) devices in non-trauma settings are limited, however, by a lack of cohort specific high-quality data [7,8].

Manchester University NHS Foundation Trust is one of two tertiary centres in the UK specialising in the surgical management of EPS. This paper presents a retrospective surgical cohort analysis comparing outcomes in OAM with static packing (betadine-soaked gauze) and NPWT in the only known study of this patient population.

In the design of this study, we hypothesised that the use of NPWT would lead to a higher rate of definitive abdominal closure and shorter time to closure compared to static packing with betadine-soaked gauze, without increasing postoperative complications.

## Materials and methods

A retrospective review of a contemporaneously maintained database of all EPS patients undergoing laparotomy between 2010 and 2021 was performed. All patients who underwent laparotomy with confirmed macroscopic EPS were included. Patients <18 years old were excluded. All patients undergoing laparotomy without primary closure had a planned relook at 24-48 hours, dependent on surgeon preference and in line with departmental protocols in place at that time.

At the end of the index operation, surgeons chose to apply a NPWT device (ABTHERA™ (3M, USA)) or apply betadine-soaked sterile abdominal gauze pads to the open abdomen. Patients who required bridged closure with mesh underwent closure with a reconstructive tissue matrix (RTM) (Strattice™ (AbbVie, Ireland)).

Primary endpoints were definitive closure, time to definitive closure, and method of closure. Method of closure was defined by fascial closure or closure with a bridged biologic mesh. Secondary endpoints included comparison of stoma formation, bowel resection, fistulation rate, enterotomy formation, re-operation post closure, intraabdominal collection, and wound infection. Failure to close was defined as inability to close the anterior fascial sheath.

Demographic and clinical data was extracted from the patient’s electronic and historic records and variables including time on peritoneal dialysis, preoperative biochemistry (including C-reactive protein (CRP) and albumin) and total duration of hospital stay were included. All patients were followed up to the time of data collection (February 2022) to ensure accurate records. A preplanned subgroup analysis of ABTHERA™ versus static packing was undertaken. Statistical analysis was performed using SPSSv.29 (IBM, USA), and figures were designed using R version 4.3.3 (Microsoft, USA) [9]. Chi-squared (χ^2^) or Fisher’s exact tests were used for categorical variables and the independent t-test or Mann-Whitney test for continuous variables. Multiple linear and logistic regression analysis was carried out to assess variables predictive of primary endpoints.

## Results

122 patients underwent laparotomy for EPS complications from 2010 to 2021 at our institution. 23 underwent primary closure at the time of their index procedure and 99 patients were managed with an open abdomen. Out of the 99 patients, 56 (57%) were managed with static packing, whilst 43 (43%) had their open abdomen managed with ABTHERA™. Both patient groups had similar baseline characteristics including age, preoperative biochemistry, and exposure to peritoneal dialysis therapy (Table 1).

**Table 1:** Clinicodemographic, operative details, and closure outcomes comparing the use of ABTHERA™ with static packing in patients undergoing surgery for EPS Clinicodemographic details were comparable between the ABTHERA™ and static packing groups with no significant differences. The static packing groups were more likely to undergo a bowel resection or stoma formation during their primary operation. The use of ABTHERA™ was associated with increased rates of fascial closure and a corresponding reduction in the need for bridged biologic closure with fewer total theatre episodes required to achieve definitive closure. Hb: Haemoglobin; CRP: C-reactive protein;  EPS: Encapsulating peritoneal sclerosis

	ABTHERA^TM ^(n=43)	Static packing (n=56)	P-Value	Statistical Test
Clinicodemographic Details				
Age (median)	49.7 (SD 18.47)	46.8 (SD 16.07)	p=0.16	Mann-Whitney
Gender (M:F)	30:13	26:30		
Time on peritoneal dialysis (months)	77.4 (SD 47.52)	86.2 (SD 51.23)	p=0.21	Mann-Whitney
Preoperative Hb (g/L)	103.4 (SD 32.55)	95.9 (SD 21.90)	p=0.09	Mann-Whitney
Preoperative albumin (g/L)	25.2 (SD 6.52)	25.2 (SD 8.67)	p=0.50	Mann-Whitney
Preoperative CRP (mg/L)	67.6 (SD 77.78)	107.6 (SD 117.96)	p=0.06	Mann-Whitney
Smoker	3 (6.9%)	4 (7.14%)	p=0.91	χ^2^
Diabetes	3 (6.9%)	8 (14.2%)	p=0.25	χ^2^
Kidney transplant recipient	24 (55.8%)	33 (58.9%)	p=0.80	χ^2^
Operative Details				
Bowel resection	9 (20.9%)	27 (48.2%)	p=0.01	χ^2^
Stoma formation	8 (14.3%)	24 (42.9%)	p=0.01	χ^2^
Enterotomies intraoperatively	9 (20.9%)	15 (26.7%)	p=0.50	χ^2^
Closure Outcomes				
Fascial closure	27 (62.8%)	7 (12.5%)	p<0.0001	χ^2^
Bridged biologic closure	14 (32.6%)	38 (67.9%)		χ^2^
Failure to close	2 (4.7%)	11 (19.6%)	p=0.002	χ^2^
Total theatre episodes (of abdomens closed)	2.27 (SD 1.03)	4.78 (SD 4.74)	p<0.0001	Mann-Whitney
Time to closure (days)	2.78 (SD 0.91)	4.62 (SD 4.42)	p=0.005	Mann-Whitney

Simple logistic regression showed factors associated with failed closure in the entire cohort (n=122) included stoma formation (p=0.014) at the time of primary operation and the postoperative occurrence of abdominal collection (p<0.0001), wound infection (p=0.0008), and fistula formation (p<0.0001). Failure to close was associated with all-cause mortality (p<0.026, χ^2^). 

Of the 86 patients who underwent successful closure after their index procedure, the only factor significantly associated with method of closure (bridged rather than fascial) was the presence of a postoperative abdominal collection (p=0.0116). There were no other significant clinicodemographic or operative associations.

Subgroup analysis: ABTHERA™ vs static packing

**Figure 1:**
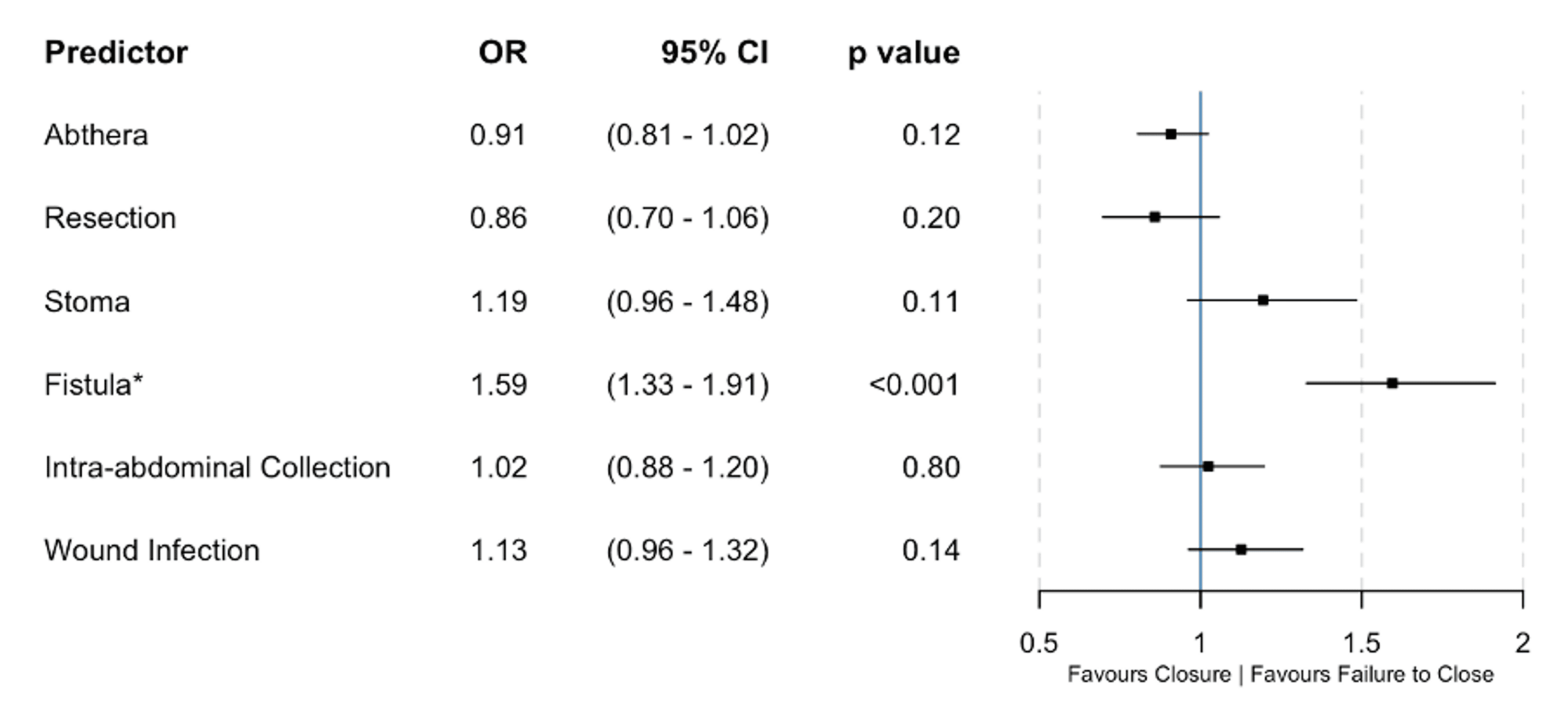
Multiple logistic regression model estimating the effect of predictors on failure to achieve definitive closure The development of a fistula is the only factor to significantly predict failure to close versus definitive fascial closure. * = Significant predictor OR: Odds ratio; CI: Confidence interval

**Figure 2:**
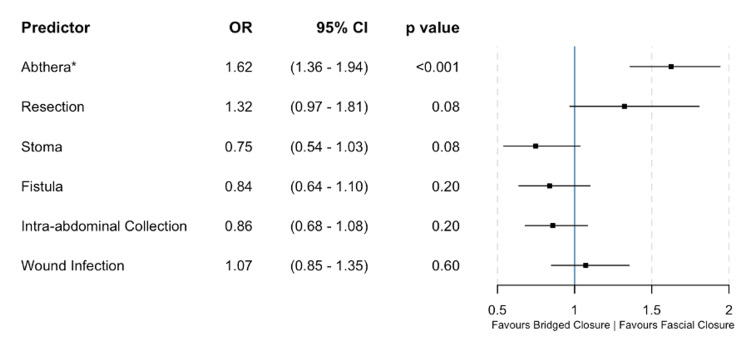
Multiple logistic regression model estimating the effect of predictors on achieving fascial closure vs bridged closure The use of ABTHERA™ rather than static packing is the only factor to predict successful primary fascial closure compared to bridged closure. * = Significant predictor OR: Odds ratio; CI: Confidence interval

63% (n=27) of patients managed with ABTHERA™ underwent fascial closure without the need for bridged or delayed closure. Patients who were managed with ABTHERA™ were more likely to undergo fascial closure when compared to those patients managed with static packing (n=27 vs n= 7, p<0.0001, χ^2^). On multiple logistic regression, fistula formation was the only parameter found to significantly predict failure to close (p<0.001, odds ratio (OR) 1.59, 95% confidence interval (CI) 1.33-1.91 (Figure 1)). However, the use of ABTHERA™ predicted fascial closure compared to bridged closure in those who did achieve a definitive repair (p<0.0001, OR 1.62, 95% CI 1.36-1.94 (Figure 2)).

The ABTHERA™ group also required less theatre episodes (n=2.27 vs. 4.78, p<0.0001, Mann-Whitney) with less failure to close episodes (p=0.002, χ^2^) and reduced time in days to achieve closure (n=2.78 vs n=4.68, p<0.005, Mann-Whitney), as demonstrated in Table 1. Multiple linear regression showed that shorter time to definitive closure was predicted by use of ABTHERA™ (p=0.035, R -1.687, R2=0.15) but not by stoma formation, bowel resection, intra-abdominal collection, wound infection, or fistulation.

Patients managed with ABTHERA™ were less likely to require a return to theatre in the 30 days post closure in comparison to the static packing group (n=4 vs n=19, p=0.004, χ^2^). The incisional hernia rate between both groups was comparable. Whilst patients in the static packing group were more likely to have a stoma or bowel resection, there was no statistical difference in the rates of enteroatmospheric fistulae documented (Table 2).

**Table 2:** Post operative complications comparing ABTHERA™ with static packing in patients undergoing surgery for EPS Static packing was associated with an increased rate of intra-abdominal collection, fistula formation and an unplanned return to theatre. EPS: Encapsulating peritoneal sclerosis

	ABTHERA^TM^ (n=43)	Static Packing (n=56)	P-Value	Statistical Test
Wound infection	6 (14%)	15 (34.9%)	p=0.12	χ^2^
Intra-abdominal collection	4 (9.3%)	22 (39.3%)	P=0.001	χ^2^
Fistula formation	3 (7%)	11 (19.6%)	p=0.07	χ^2^
Unplanned return to theatre (30 days)	4 (9.3%)	19 (16.1%)	p=0.004	χ^2^
Incisional hernia	6 (14.6%)	7 (13.3%)	p=0.86	χ^2^

23% (n=10) of patients managed with ABTHERA™ underwent colonic resection and/or stoma formation.

There was no statistically significant difference in time to closure (p=0.4, Mann-Whitney) or in primary fascial closure (p>0.9, Fisher’s exact test) in this subgroup, nor in any other outcome (Table 3).

**Table 3:** Outcomes following ABTHERA™ use in patients requiring bowel resection +/- stoma in patients undergoing surgery for EPS In patients undergoing bowel resection or stoma formation, ABTHERA™ use has comparable outcomes compared to patients without resection/stoma, demonstrating its safety. EPS: Encapsulating peritoneal sclerosis

ABTHERA^TM^ (n=43)	Resection/Stoma (n=10)	No Resection/Stoma (n=33)	P-Value	Statistical Test
Wound infection	2 (20%)	3 (9.1%)	0.6	χ^2^
Intra-abdominal collection	0 (0%)	4 (12%)	0.6	χ^2^
Fistula formation	1 (10%)	2 (6.1%)	>0.9	χ^2^
Fascial closure	6 (60%)	21 (64%)	>0.9	χ^2^
Failure to close	1 (10%)	0 (0%)	0.2	χ^2^
Time to closure (days)	2.5 (SD 0.53)	2.84 (SD 0.99)	0.4	Mann-Whitney
90-day mortality	1 (10%)	2 (6%)	>0.9	χ^2^

## Discussion

ABTHERA™ is an abdominal closure technique with a visceral protective layer (VPL), a non-adherent fenestrated polyurethane dressing, and a perforated foam that delivers negative pressure therapy, with the additional benefit of minimising fascial retraction [8]. Use of ABTHERA™ in OAM has been associated with higher fascial closure rates (of up to 89%) with lower all cause 30-day mortality. This is proposed to be due to the impact of reduced inflammatory cytokine burden from negative pressure mediated drainage of peritoneal fluid, and the indirect impact on reduced tissue oedema [10,11]. It has long been established that ABTHERA™ contributes to a reduction in loss of abdominal domain by supporting abdominal wall tension to reduce fascial retraction [10-12]. Mortality associated with OAM can exceed 30%, and there is an increased association with prolonged duration of open abdomen [10,13,14]. Fascial closure rates in this series are comparable to those reported elsewhere in the literature. In our cohort, 74% (n=32) of patients managed with ABTHERA™ had successful primary or bridged closure within 72 hours, with 100% (n=41) closed at five days. The patients managed with static packing in which primary or delayed closure was achieved had a 25% (n=11) rate of remaining open at five days.

Our patient cohort represented a homogenous group of chronically malnourished patients, the majority of whom had undergone at least one kidney transplant. These patients had a heavily sclerosed peritoneum, which contributed significantly to increased stiffness of the abdominal wall. Due to the insidious nature of EPS, many patients presented as acute surgical emergencies significantly deconditioned with advanced intraperitoneal disease, which placed them at a higher risk of suboptimal outcomes [3]. Despite these patient characteristics, the use of ABTHERA™ was associated with an increased likelihood of achieving fascial closure and shorter time to definitive abdominal closure, as well as reduced risk of re-operation at 30 days post closure.

There has been longstanding concern about the use of NPWT and its association with the development of enteroatmospheric fistulae because of direct contact with intestinal loops [15-17]. The development of newer technologies, such as ABTHERA™, has superseded these perceptions through the incorporation of a VPL to its device [18,19]. Our low enteroatmospheric fistula rate is comparable to other published papers using NPWT with VPLs quoting fistula rates of 3.5-4% and offers further reassurance of it’s efficacy in the emergent setting [20,21]. In a recent propensity matched analysis of the European Hernia Society Open Abdomen Registry, the use of VPL wound management devices led to significantly lower fistulation rates when compared to a control group [22].

Current consensus guidelines published by the Association of Coloproctology of Great Britain and Ireland (ACPGBI) recognise the limited evidence base of OAM in non-trauma settings [23,24]. They advocate for the appropriate selective use of vacuum-assisted closure with mesh-mediated traction as an optimal strategy to minimise complications. The lack of consensus guidelines may in part lead to significant variations in practice, therefore impacting the accuracy and reproducibility of reported patient outcomes, an inherent limitation in any similar study. A large retrospective series demonstrated higher rates of OAM in a cohort of laparotomies performed at night by non-subspecialist surgeons and concurrently reported higher rates of postoperative complications, mortality, and inpatient stay [25]. At our institution, we have now established the use of ABTHERA™ as standard operating procedure in this patient cohort with a recognised focus on early and planned closure, which is reflected in our low complication rates [26].

We have demonstrated in this study that the need to form a stoma or perform a bowel resection does not preclude the use of NPWT, which has incorrectly historically been a limiting factor in its use [18]. In this series, all but one patient managed with ABTHERA™ that required a resection and/or stoma underwent primary or bridged closure within 72 hours (Table 3). Similarly, there was no significant difference in the decision to apply ABTHERA™ or betadine-soaked gauze in the setting of enterotomies identified at the time of surgery. Limited extrapolated data is available regarding the use of NPWT in the setting of bowel resections. In a retrospective cohort study comparing use of ABTHERA™ with abdominal dressings, there was no significant difference in fistula rates following bowel resection [21]. In more recent a propensity matched analysis evaluating the role of NPWT with VPL in secondary peritonitis, there were significantly less complications associated with VPL accounting for a range of pathologies associated with secondary peritonitis [27]. A strength of this study is our homogenous patient cohort, characterised often by severe cases of obstruction and peritonitis, which adds to the growing body of evidence of NPWT being effectively applied in non-trauma settings.

It could be considered that those patients deemed too high risk for NPWT at the time of index laparotomy in our cohort were preferentially selected for packing with betadine-soaked gauze, creating an inherent bias. However, analysis of patient characteristics demonstrates similar attributes except for a non-significant difference in CRP levels between groups (Table 1). In reviewing the method of abdominal closure in our dataset from 2017, only two patients were managed with static packing, compared to 24 patients who were managed with ABTHERA™, which represents a move towards an established institutional standard of care for OAM in recent years. In this subset of patients, four of the 10 ABTHERA™ patients underwent colonic resection with or without stoma formation. Additionally, there were six patients with enterotomies identified intraoperatively, all of whom were managed with ABTHERA™.

We recognise the limitations and potential reporting bias of this retrospective review. There have been calls internationally to establish clinical equipoise in the management of open abdomen in non-trauma settings. The Closed or Open Laparotomy (COOL) study to evaluate open abdomen management in secondary peritonitis is an ongoing multicentre randomised controlled trial currently ongoing but there have been challenges with recruitment [28].

## Conclusions

In conclusion, we have demonstrated that the use of NPWT is associated with increased likelihood of fascial closure with a reduced complication profile in a chronically malnourished, high risk surgical cohort. Whilst this analysis focuses on a highly sub-specialised surgical practice, we propose that our experience and results with ABTHERA™ can be generalised to those chronically malnourished or extremely co-morbid patients who would benefit from a reduction in the number of surgical interventions and concomitant general anaesthesia. This study adds to a growing body of evidence for the effective application of NPWT in non-trauma settings, for example in settings of sepsis, burst abdomen, or in which the postoperative outlook is uncertain at the time of initial or relook surgery. In this retrospective cohort analysis, we have demonstrated that NPWT is associated with increased likelihood of fascial closure with a reduced complication profile. Additionally, we have demonstrated its safe application in the presence of bowel resections and stomas. We propose that ABTHERA™ should be added to the armamentarium of the general surgeon in managing the high-risk open abdomen.

## References

[1] Danford CJ, Lin SC, Smith MP, Wolf JL (2018). Encapsulating peritoneal sclerosis. World J Gastroenterol.

[2] Augustine T, Brown PW, Davies SD, Summers AM, Wilkie ME (2009). Encapsulating peritoneal sclerosis: clinical significance and implications. Nephron Clin Pract.

[3] Moinuddin Z, Summers A, Van Dellen D, Augustine T, Herrick SE (2015). Encapsulating peritoneal sclerosis-a rare but devastating peritoneal disease. Front Physiol.

[4] Brown MC, Simpson K, Kerssens JJ, Mactier RA (2009). Encapsulating peritoneal sclerosis in the new millennium: a national cohort study. Clin J Am Soc Nephrol.

[5] Kawanishi H (2012). Surgical and medical treatments of encapsulation peritoneal sclerosis. Contrib Nephrol.

[6] Latus J, Ulmer C, Fritz P (2013). Encapsulating peritoneal sclerosis: a rare, serious but potentially curable complication of peritoneal dialysis-experience of a referral centre in Germany. Nephrol Dial Transplant.

[7] Coccolini F, Roberts D, Ansaloni L (2018). The open abdomen in trauma and non-trauma patients: WSES guidelines. World J Emerg Surg.

[8] Atema JJ, Gans SL, Boermeester MA (2015). Systematic review and meta-analysis of the open abdomen and temporary abdominal closure techniques in non-trauma patients. World J Surg.

[9] (2025). The R Project for Statistical Computing. https://www.R-project.org.

[10] Coccolini F, Biffl W, Catena F (2015). The open abdomen, indications, management and definitive closure. World J Emerg Surg.

[11] Lindstedt S, Malmsjö M, Hlebowicz J, Ingemansson R (2015). Comparative study of the microvascular blood flow in the intestinal wall, wound contraction and fluid evacuation during negative pressure wound therapy in laparostomy using the V.A.C. abdominal dressing and the ABThera open abdomen negative pressure therapy system. Int Wound J.

[12] Cheatham ML, Demetriades D, Fabian TC (2013). Prospective study examining clinical outcomes associated with a negative pressure wound therapy system and Barker's vacuum packing technique. World J Surg.

[13] Carlson GL, Patrick H, Amin AI (2013). Management of the open abdomen: a national study of clinical outcome and safety of negative pressure wound therapy. Ann Surg.

[14] Miller RS, Morris JA Jr, Diaz JJ Jr, Herring MB, May AK (2005). Complications after 344 damage-control open celiotomies. J Trauma.

[15] Rao M, Burke D, Finan PJ, Sagar PM (2007). The use of vacuum-assisted closure of abdominal wounds: a word of caution. Colorectal Dis.

[16] Bee TK, Croce MA, Magnotti LJ (2008). Temporary abdominal closure techniques: a prospective randomized trial comparing polyglactin 910 mesh and vacuum-assisted closure. J Trauma.

[17] Fischer JE (2008). A cautionary note: the use of vacuum-assisted closure systems in the treatment of gastrointestinal cutaneous fistula may be associated with higher mortality from subsequent fistula development. Am J Surg.

[18] Bobkiewicz A, Walczak D, Smoliński S (2017). Management of enteroatmospheric fistula with negative pressure wound therapy in open abdomen treatment: a multicentre observational study. Int Wound J.

[19] Fernández LG (2016). Management of the open abdomen: clinical recommendations for the trauma/acute care surgeon and general surgeon. Int Wound J.

[20] Hougaard HT, Ellebaek M, Holst UT, Qvist N (2014). The open abdomen: temporary closure with a modified negative pressure therapy technique. Int Wound J.

[21] Olona C, Caro A, Duque E, Moreno F, Vadillo J, Rueda JC, Vicente V (2015). Comparative study of open abdomen treatment: ABThera™ vs. abdominal dressing™. Hernia.

[22] Schaaf S, Schwab R, Wöhler A (2023). Use of a visceral protective layer prevents fistula development in open abdomen therapy: results from the European Hernia Society Open Abdomen Registry. Br J Surg.

[23] Miller AS, Boyce K, Box B (2021). The Association of Coloproctology of Great Britain and Ireland consensus guidelines in emergency colorectal surgery. Colorectal Dis.

[24] Schembari E, Richardson C, King AT, Layfield DM (2024). Mesh mediated fascial traction in the management of the open abdomen: a video vignette. Colorectal Dis.

[25] Kao AM, Cetrulo LN, Baimas-George MR, Prasad T, Heniford BT, Davis BR, Kasten KR (2019). Outcomes of open abdomen versus primary closure following emergent laparotomy for suspected secondary peritonitis: a propensity-matched analysis. J Trauma Acute Care Surg.

[26] Slade DA (2024). Open abdomen in secondary peritonitis: time for closure. Br J Surg.

[27] Willms AG, Schaaf S, Zimmermann N (2021). The significance of visceral protection in preventing enteroatmospheric fistulae during open abdomen treatment in patients with secondary peritonitis: a propensity score-matched case-control analysis. Ann Surg.

[28] Kirkpatrick AW, Coccolini F, Tolonen M (2023). The unrestricted global effort to complete the COOL trial. World J Emerg Surg.

